# A Non-Enzymatic Sensor Based on Trimetallic Nanoalloy with Poly (Diallyldimethylammonium Chloride)-Capped Reduced Graphene Oxide for Dynamic Monitoring Hydrogen Peroxide Production by Cancerous Cells

**DOI:** 10.3390/s20010071

**Published:** 2019-12-21

**Authors:** Jun Jiao, Meixin Pan, Xinran Liu, Binshuai Li, Jian Liu, Qiang Chen

**Affiliations:** The Key Laboratory of Bioactive Materials Ministry of Education, College of Life Science, Nankai University, Tianjin 300071, China

**Keywords:** non-enzymatic biosensors, nanosheets, trimetallic nanoalloy, reactive oxygen species, living cancer cells

## Abstract

Catching cancer at an early stage is necessary to make it easier to treat and to save people’s lives rather than just extending them. Reactive oxygen species (ROS) have sparked a huge interest owing to their vital role in various biological processes, especially in tumorigenesis, thus leading to the potential of ROS as prognostic biomarkers for cancer. Herein, a non-enzymatic biosensor for the dynamic monitoring of intracellular hydrogen peroxide (H_2_O_2_), the most important ROS, via an effective electrode composed of poly (diallyldimethylammonium chloride) (PDDA)-capped reduced graphene oxide (RGO) nanosheets with high loading trimetallic AuPtAg nanoalloy, is proposed. The designed biosensor was able to measure H_2_O_2_ released from different cancerous cells promptly and precisely owing to the impressive conductivity of RGO and PDDA and the excellent synergistic effect of the ternary alloy in boosting the electrocatalytic activity. Built upon the peroxidase-like activity of the nanoalloy, the developed sensor exhibited distinguished electrochemical performance, resulting in a low detection limit of 1.2 nM and a wide linear range from 0.05 μM to 5.5 mM. Our approach offers a significant contribution toward the further elucidation of the role of ROS in carcinogenesis and the effective screening of cancer at an early stage.

## 1. Introduction

Cancer is a global issue and has a major impact on public health. It is estimated that there were 18.1 million new cancer cases and 9.6 million deaths from cancer in 2018 around the world [1]. As the technology evolves, researchers are focusing on ways to detect, treat, and prevent cancer earlier to level off or decrease the incidence of and mortality due to cancer. Although cancer remains one of the leading causes of death, diagnosing it early offers the opportunity to improve survival rates [2]. The broader application of advanced techniques like X-ray imaging, computed tomography (CT), positron emission tomography (PET), and biopsy in cancer diagnosis has accelerated progress against cancer [3,4,5]. However, the accuracy of such typical methods is reduced if carcinogens are present at the very beginning. The sooner cancer is diagnosed, the higher the survival rate will be. Therefore, if identifying precancerous lesions or tumors in the early initiation phase can be accomplished, there is more chance of administering more precise therapies.

It has been proven that reactive oxygen species (ROS), including superoxide (O^2−^), hydrogen peroxide (H_2_O_2_), hydroxyl radicals, and peroxynitrite, are messenger molecules, which can turn various biological processes on and off [6]. Balanced ROS metabolism increases antioxidant ability, thereby establishing a barrier against tumorigenesis [7,8]. However, DNA damage caused by ROS results in genomic instability and altered gene expression, while ROS-mediated signaling pathways also drive cell proliferation and apoptosis suppression, thus promoting tumor formation [9,10]. Among the ROS, H_2_O_2_ has sparked huge interest in anticancer therapies as not only is it the main substance in the cellular response to oxidative stress, but it is also able to pass through cell membranes over a relatively long lifetime. In addition, cancer cells have metabolic and signaling aberrations, and thus exhibit an enhanced H_2_O_2_ level that differs from that in normal cells [11,12]. Therefore, the measurement of intracellular H_2_O_2_ could be of great value in clarifying its role in oncogenesis and exploring advanced therapeutic strategies for cancer and other ROS-related diseases.

Although various analytical techniques for measuring H_2_O_2_, such as spectrophotometry and chemiluminescence, have been developed, they still suffer drawbacks such as low detection limits and complex processing when faced with the challenge of achieving detection limits in the nanomolar range in living cells [13,14]. In this regard, electrochemical techniques with high sensitivity and simplicity to improve the timely detection of H_2_O_2_ in vivo have gained increasing attention. Enzymes like horseradish peroxidase have been widely used for biosensor fabrication as they own impressive specificity and notable sensitivity towards target analytes [15,16,17]. Dai et al. [18] immobilized horseradish peroxidase (HRP) on a boronic acid-functionalized metal–organic framework to fabricate a sensitive sensor for the real-time detection of H_2_O_2_ released from living cells. However, such enzymatic sensors have been gradually replaced by non-enzymatic ones owing to their deficiency in enzyme immobilization and instability [19,20,21]. Noble metals, alloys, and metal oxides have played remarkable roles as robust inorganic catalysts in non-enzymatic biosensors for bimolecular detection on account of their intrinsic peroxidase-like activity [22,23,24,25,26]. For instance, platinum revealed admirable catalytic activity for reduction of hydrogen peroxide, which effectively enhances the analytical performances of biosensors [27,28]. Muhammet et al. reported a non-enzymatic biosensor with outstanding electrochemical properties by decorating Pd@Au bimetallic nanoparticles and reduced graphene oxide on the surface of electrode for determination of H_2_O_2_. Meanwhile, the constructed sensor achieved the detection in a real milk sample [29]. Compared with their bimetallic or monometallic counterparts, trimetallic catalysts with an enlarged surface area, abundant active sites, and fast mass transfer present a pronounced synergistic effect, resulting in superior electrocatalytic activity and stability [30,31]. Hence, trimetallic catalysts hold considerable promise as competitive candidates for the real-time cellular monitoring of ROS levels after oxidative stress. Reduced graphene oxide (RGO) is a widely used competitor for the construction of sensing interfaces by virtue of its great electrical conductivity, its extensive surface area, and the fact that it is conveniently functionalizable [32,33,34]. However, it has a strong inclination to agglomerate irreversibly owing to van der Waals interactions, leading to poor dispersibility in aqueous solutions [35]. The cationic polymer poly (diallyldimethylammonium chloride) (PDDA) was introduced to tackle this problem as the electrostatic charge in RGO can be altered by PDDA, thus avoiding the agglomeration of RGO and revealing its more engaging characteristics such as hydrophilicity and modifiability [36,37].

**Scheme 1:**
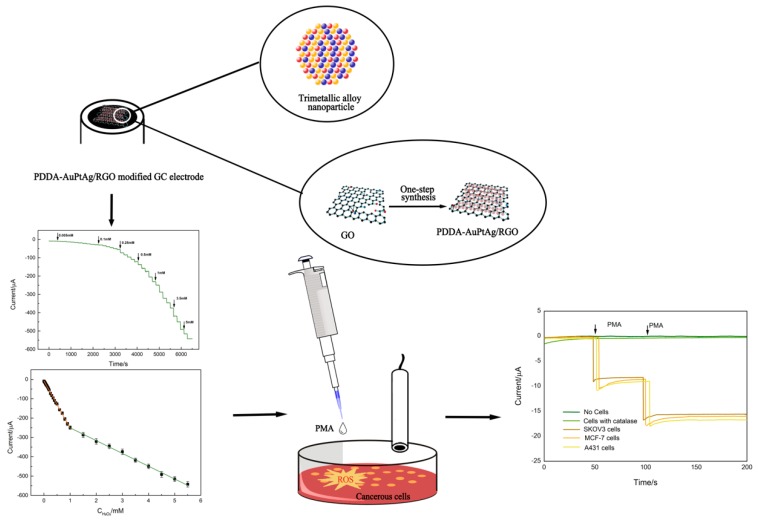
Schematic illustration for the preparation of poly (diallyldimethylammonium chloride) (PDDA)-AuPtAg/reduced graphene oxide (RGO)-modified glassy carbon electrode (GCE) applied for the detection of H_2_O_2_ released from SKOV3, MCF-7, and A431 cells stimulated with phorbol-12-myristate-13-acetate (PMA). ROS, reactive oxygen species.

In this work, we utilized a one-pot and cost-effective method for synthesizing PDDA-capped AuPtAg/RGO nanohybrids. The obtained nanocomposites presented stronger catalytic and conductive capacity via incrementing the electroactive surface area and enhancing the electron transport efficiency. By analyzing the electrochemical responses from the PDDA–RGO nanocomposites and nanoalloy particles, the dynamic levels of H_2_O_2_ in different living cancerous cells were determined almost instantly with satisfactory results (Scheme 1). The as-prepared sensor exhibited a limit of detection (LOD) as low as 1.2 nM, thus indicating its usefulness as a platform for monitoring differences in H_2_O_2_ in cancerous tissues and opening the door to identify its role in tumor formation.

## 2. Materials and Methods

### 2.1. Reagents and Apparatus

Graphene oxide (GO) was acquired from Nanjing XFNANO Materials Tech Co. (China). Platinic chloride hydrate, hydrogen tetrachloroaurate trihydrate (HAuCl_4_·3H_2_O, 99%), silver nitrate, poly (diallyldimethylammonium chloride) (PDDA), glucose, ascorbic acid (AA), uric acid (UA), xanthine, and phorbol-12-myristate-13-acetate (PMA) to liberate H_2_O_2_ from live cells were received from Sigma Aldrich Co. Cell culture medium (Dulbecco’s modified essential medium (DMEM), McCoy’s 5A) and Amplex Red Hydrogen Peroxide Assay kit was purchased from Thermo Fisher. These were used as such without any further purification. All working aqueous solutions were prepared with double distilled water.

All electrochemical measurements were conducted using 283 Potentiostat-Galvanostat electrochemical workstation (EG&GPARC with M270 software) with an ordinary triple-electrode system. It consisted of an Ag/AgCl electrode saturated with KCl, a platinum wire, and a modified glassy carbon electrode as the reference, counter, and adopted working electrode, respectively. All experiments were performed at room temperature. In addition, transmission electron microscopy (TEM) image analysis was obtained from Tecnai G2 F20 instrument (Philips Holland), while an energy-dispersive X-ray spectroscopy (EDX) analyzer on the Tecnai G2 F20 carried out the EDX spectrum and mapping analysis. The X-ray diffraction (XRD) patterns were performed on Rigaku D/max-rA (Rigaku, Japan) using Cu Kα radiation (λ = 1.5418 Å).

### 2.2. One-Pot Synthesis of PDDA-AuPtAg/RGO Nanohybrids

Firstly, 20 mg of GO was dispersed in 20 mL double distilled water containing 1% poly (diallyldimethylammonium chloride) (PDDA) solution followed by 2 h ultrasonication for the formation of homogeneous PDDA–GO aqueous solution. Then, HAuCl_4_ solution (100 μL, 1 M), H_2_PtCl solution (100 μL, 1 M), and 10 mg AgNO_3_ in 20 mL double distilled water were mixed with PDDA–GO aqueous solution. Then, 10 mL NaBH_4_ solution was added dropwise under magnetic stirring at room temperature. With constant stirring lasting 24 h, the reaction was terminated until dark black products formed. Subsequently, PDDA-AuPtAg/RGO nanoalloy was obtained after centrifugation and washing by ethanol three times. Finally, the product was dried in a vacuum oven at 80 °C and prepared for use. RGO, PDDA-AuPt/RGO, PDDA-AuAg/RGO, and PDDA-PtAg/RGO were prepared through using a similar method.

### 2.3. Preparation of Modified Electrodes

The glassy carbon (GC) electrode was polished with 0.3 and 0.05 μm α-alumia powder before modification, and then washed by double distilled water and ethanol, respectively, followed by drying with high-purity nitrogen stream. Then, 10 mg of PDDA-AuPtAg/RGO was re-dispersed in 10 mL of double distilled water with sonication for 2 h, after which 6 μL of the suspension was immobilized on the surface of the GC electrode by dip-coating and dried in air. 

### 2.4. Real-Time Detection of H_2_O_2_ Released From Living Cells

The cells tested in this work were SKOV3 cells (human ovarian cancer cell line), MCF-7 cells (breast cancer cell line), and A431 cells (epidermoid carcinoma cell line) obtained from School of Basic Medical Science, Tianjin Medical University, Tianjin, China (come from ATCC). SKOV3 cells and A431 cells were maintained in McCoy’s 5A (Modified) Medium and DMEM containing 10% fetal bovine serum, respectively. For MCF-7 cells, they were maintained in Dulbecco’s modified essential medium (DMEM) containing 10% fetal bovine serum, 0.01 mg/ml human recombinant insulin, 100 units/mL penicillin, and 100 μg/mL streptomycin. All the cells were incubated under ambient conditions with 37 °C, 5% CO_2_, 95% humidified atmosphere, and sub-cultured after every second day.

After sub-cultured to 90% confluence, the cells were washed three times with phosphate-buffered saline (PBS) (0.1 M, pH 7.0) and collected by centrifugation. The cell number was calculated by using a cell counter. A total of 1 × 106 cells were resuspended into 10 mL 0.1 M PBS (pH 7.0) as cell samples and the control group containing catalases (300 U/mL) was also prepared. For electrochemical measurements, injection of 10 μL PMA (1 μg mL^−1^) was applied into the tested system and current responses on the PDDA-AuPtAg/RGO/GCE were recorded at the potential of 0.13 V, as shown in Scheme 1.

## 3. Results and Discussion

### 3.1. Characterization of PDDA-AuPtAg/RGO Nanocomposites

**Figure 1:**
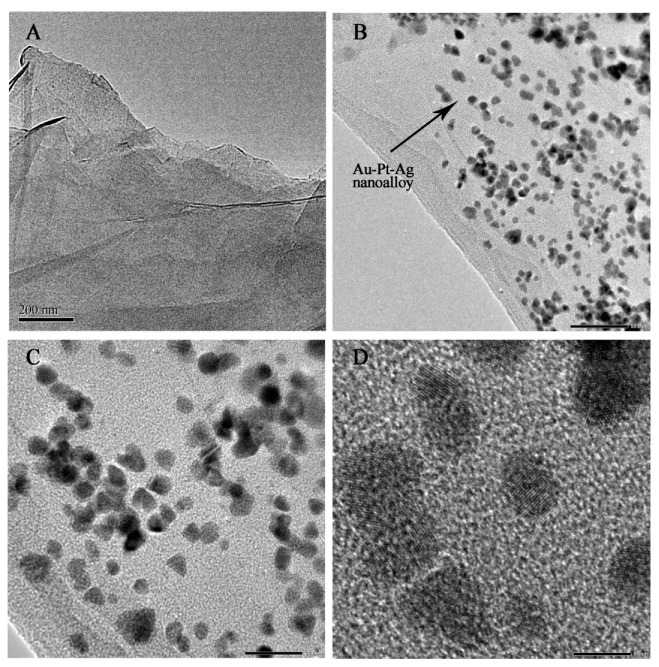
Transmission electron microscopy (TEM) images of (**A**) reduced graphene oxide (RGO) and (**B**–**D**) poly (diallyldimethylammonium chloride) (PDDA)-AuPtAg/RGO under different magnifications.

**Figure 2:**
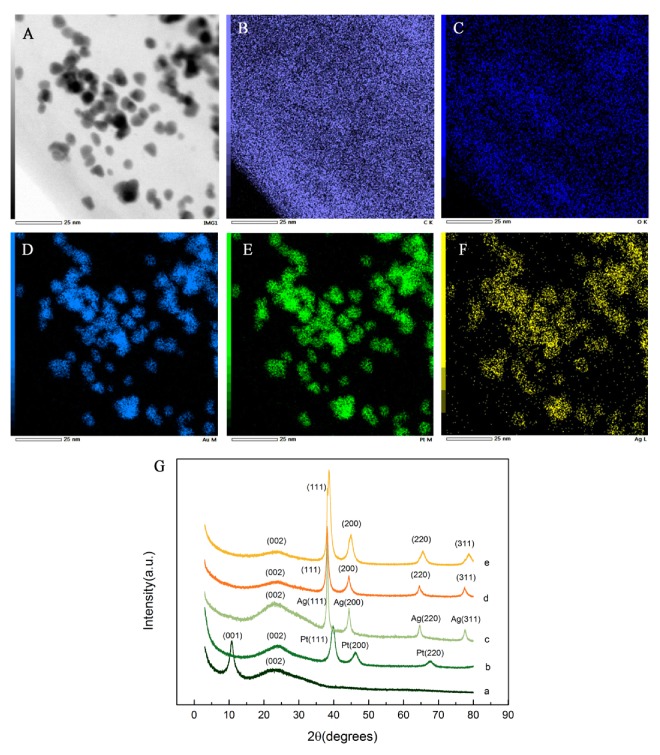
TEM elemental mapping for (**A**) PDDA-AuPtAg/RGO and (**B**) C, (**C**) O, (**D**) Au, (**E**) Pt, and (**F**) Ag in the nanocomposite. (**G**) X-ray diffraction (XRD) spectra of (a) GO, (b) PDDA-Pt/RGO, (c) PDDA-Ag/RGO, (d) PDDA-AuAg/RGO, and (e) PDDA-AuPtAg/RGO.

To identify the morphology of the as-prepared nanocomposites, transmission electron microscopy (TEM) was used for structural analysis. In Figure 1A, a typical wrinkled pattern of reduced graphene oxide sheets can be observed. Under different magnification (Figure 1B,C), it can be clearly seen that AuPtAg alloy nanoparticles (as pointed out in Figure 2B) distributed well on the PDDA–RGO sheets with less aggregation owing to excellent dispersing performance of poly dimethyl diallyl ammonium chloride (PDDA). The PDDA-AuPtAg alloy nanocrystals displayed paralleled lattice fringes along the same orientation (Figure 1D), indicating there were single AuPtAg nanoalloy particles locating on the PDDA-capped reduced graphene oxide layers.

A TEM elemental mapping analysis (Figure 2A–F) and energy dispersive X-ray (EDX) spectroscopy (Figure S2) were conducted to verify the components of the PDDA-AuPtAg/RGO composites. The results indicate that the obtained nanocomposites consisted of C, O, Au, Pt, and Ag. The C and O elements were evenly distributed as a film, while Au, Pt, and Ag were relatively concentrated, implying the formation of a trimetallic alloy. Figure 2G shows X-ray diffraction (XRD) spectra of GO, PDDA-Pt/RGO, PDDA-Ag/RGO, PDDA-AuAg/RGO, and PDDA-AuPtAg/RGO. GO presented a typical XRD diffraction pattern with a sharp peak at around 10° and a wider peak at 23.99°. The peak at 10° disappeared with the formation of the various nanocomposites, thus indicating that GO was reduced to RGO [38]. The diffraction peaks at around 39.8°, 46.3°, and 67.8° for PDDA-Pt/RGO were associated with the (111), (200), and (220) facets of the Pt nanoparticles, respectively [39,40]. In addition, peaks at 38.1°, 44.1°, 64.4°, and 77.3° for PDDA-Ag/RGO were attributed to the reflections on the (111), (200), (220), and (311) planes of Ag, respectively. Meanwhile, the characteristic peaks at 38.3°, 44.3°, 64.9°, and 77.69° for the bimetallic PDDA-AuAg/RGO were indexed to diffraction peaks of the (111), (200), (220), and (311) lattice planes of Au, respectively, according to Bragg’s reflection with no obvious diffraction peaks of silver, indicating that the surfaces of the Ag nanoparticles were replaced with Au, which led to the formation of a core–shell structure [41]. Compared with its counterparts, all of the peaks in the diffraction pattern for PDDA-AuPtAg/RGO were broader owing to the superposition of lattice planes, which suggests the formation of a trimetallic alloy composite.

### 3.2. Electrochemical Behavior of Obtained Materials

**Figure 3:**
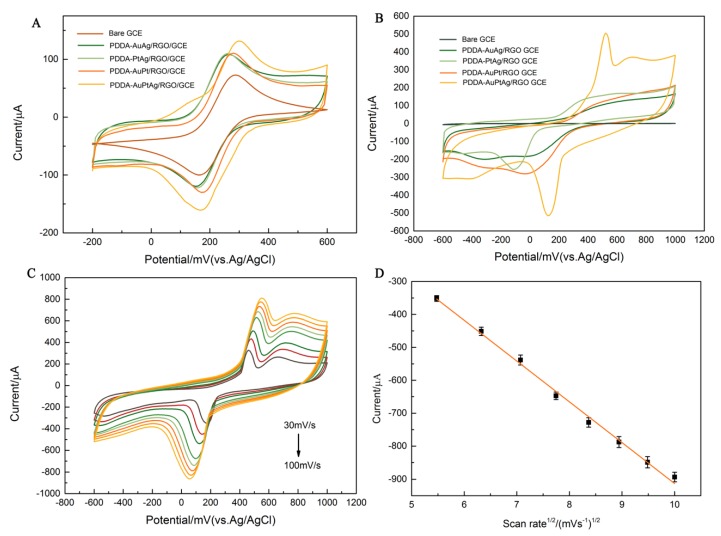
Cyclic voltammograms (CVs) for bare glassy carbon electrode (GCE) (red), PDDA-AuAg/RGO/GCE (green), PDDA-PtAg/RGO/GCE (light green), PDDA-AuPt/RGO/GCE (orange), and PDDA-AuPtAg/RGO/GCE (yellow) recorded in (**A**) 0.1 M KCL solution containing 10 mM [Fe (CN)_6_]^3–^ and (**B**) 0.1 M phosphate-buffered saline (PBS) (pH 7.4) in the presence of 5 mM H_2_O_2_ at a scan rate of 50 mV/s. (**C**) CVs for PDDA-AuPtAg/RGO/GCE with 5 mM H_2_O_2_ in 0.1 M PBS (pH 7.0) at scan rates of 30, 40, 50, 60, 70, 80, 90, and 100 mV/s. (**D**) A plot of the cathodic peak current vs. v^1/2^.

Electrochemical characterization using ferro/ferricyanide as the redox probe was accomplished in cyclic voltammograms (CVs) for a bare glassy carbon electrode (GCE), PDDA-AuAg/RGO, PDDA-PtAg/RGO, PDDA-AuPt/RGO, and PDDA-AuPtAg/RGO, modified GCE electrodes (Figure 3A). PDDA-AuPtAg/RGO/GCE revealed a pair of well-defined redox peaks at around +300 mV and +172 mV. Compared with bare GCE and the modified GCE electrodes with bimetal composites, an obvious increment in the peaks was observed for PDDA-AuPtAg/RGO/GCE owing to its fast electron-transfer kinetics. To further characterize the obtained material, microscopic electroactive areas were estimated according to the Randles–Sevcik equation [42]:

(1) $$Ip=2.69\times 10^{5}\times A\times D^{1/2}\times n^{3/2}\times v^{1/2}\times c,$$

where I*_p_* relates to the redox peak current, *A* corresponds to the electroactive surface area (cm^2^), the diffusion coefficient (*D*) of the molecules in solution is (6.70 ± 0.02) × 10^−6^ cm^2^/s, *n* represents the number of electrons participating in the redox reaction (equal to 1), *v* is the scan rate (V/s), and *c* is the bulk concentration of the redox probe (mol cm^3^). According to Equation (1), the value of *A* for PDDA-AuPtAg/RGO/GCE was calculated as 0.086 cm^2^, which was 1.72, 1.45, 1.34, and 1.21 times larger than unmodified GCE, PDDA-AuAg/RGO/GCE, PDDA-PtAg/RGO/GCE, and PDDA-AuPt/RGO/GCE, respectively. These results prove that the PDDA-capped trimetallic alloy with RGO nanosheets with an enlarged surface area was more applicable for accelerating the electron transfer between K_3_[Fe (CN)_6_] and the working electrode.

### 3.3. Electrochemical Response to H_2_O_2_ by PDDA-AuPtAg/RGO/GCE

The electrocatalytic performance of PDDA-AuPtAg/RGO/GCE toward H_2_O_2_ was investigated in the presence of 5 mM H_2_O_2_, the results of which are shown in Figure 3B. A distinctly higher current response at around 0.13 V/0.72 V was attained by fabricating PDDA-AuPtAg/RGO on the GCE. In contrast, there was no response on bare GCE and the current changes of bimetal counterparts were much lower, suggesting that the trimetallic alloy composite responded well to the reduction of H_2_O_2_. The remarkable improvements of response signals could be contributed to the outstanding peroxidase-like activity of single Au, Pt, and Ag nanoparticles and the synergistic effects after integration [43,44,45]. Besides this, PDDA–RGO nanosheets also play a compelling part in boosting the performance of the proposed electrode, offering more arching sites for trimetallic alloy nanocomposites as a supporting platform. Therefore, the electrode modified with PDDA-AuPtAg/RGO exhibited excellent catalytic activity toward the reduction of H_2_O_2_.

### 3.4. Performance of the Proposed Sensor

#### 3.4.1. Optimization of the Experimental Variables

As shown in Figure 3C,D, typical CV was adopted to investigate the effects of the scan rate at PDDA-AuPtAg/RGO/GCE. It can be clearly observed that the oxidation and reduction currents for H_2_O_2_ increased linearly along with the rising scan rate. In addition, there was a clear linear relationship between the redox peak current and the square root of scan rate (v^1/2^) using the regression equation Ip (μA) = −111.17υ (mV/s) + 147.84 (R^2^ = 0.997), indicating a diffusion-controlled process [46]. Other experimental parameters including concentration and deposition volume of PDDA-AuPtAg/RGO and pH were also investigated to determine the optimal sensor response (Figure S2, S3). In summary, the PDDA-AuPtAg/RGO/GCE demonstrated the best response performance toward H_2_O_2_ under the following conditions: 6 μL of PDDA-AuPtAg/RGO (1 mg/mL), phosphate-buffered saline (PBS) at PH 7.0, and a scan rate of 50 mV/s.

#### 3.4.2. Amperometric Response towards H_2_O_2_

**Figure 4:**
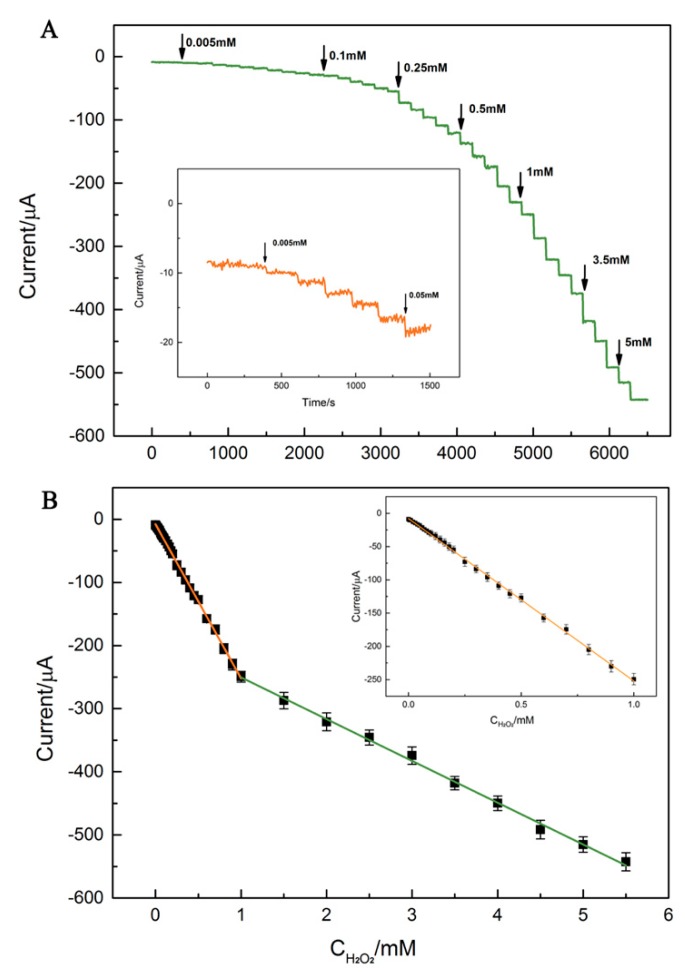
(**A**) Amperometric i-t curves for PDDA-AuPtAg/RGO/GCE at 0.13 V (vs. Ag/AgCl) in 0.1 M PBS (pH 7.0) with successive additions of H_2_O_2_ (the insert shows a close-up of the low concentration of H_2_O_2_ region from 0.005 mM to 0.05 mM). (**B**) The corresponding calibration curve of response current vs. the concentration of H_2_O_2_.

The constructed sensor based on PDDA-AuPtAg/RGO was used to determine the H_2_O_2_ concentration via amperometric i-t curves under optimal conditions. Figure 4A shows the amperometric response toward different concentrations of H_2_O_2_. In pace with the rising concentration of H_2_O_2_, the current response increased gradually, with 95% of the steady-state current being achieved within 5 s. The calibration curve (Figure 4B) was fitted after five independent repetitive experiments and the PDDA-AuPtAg/RGO modified GCE revealed an exceptional linear relationship between current response and H_2_O_2_ concentration in two segments: from 0.05 μM to 1 mM and from 1 mM to 5.5 mM. As shown in the insert in Figure 4B, the corresponding linear equation is I = −244.15C − 7.83 (R^2^ = 0.9985) with a sensitivity of 2838.95 μA·mM^−1^·cm^−2^ in the range from 0.05 μM to 1 mM. In the meantime, another linear relationship, I = −66.18C − 184.12 (R^2^ = 0.9978), existed from 1 mM to 5.5 mM. The calculated detection limit for H_2_O_2_ was 1.2 nM at a signal-to-noise ratio of 3. These results suggest that the PDDA-AuPtAg/RGO fabricated sensor achieved a wide detection range and a low detection limit when determining H_2_O_2_ concentration, thus showing it to be a credible and powerful platform for monitoring cellular H_2_O_2_ levels.

In addition, a comparison on the performance of our designed sensor with previously reported H_2_O_2_ biosensors is summarized in Table S1, in which it can be clearly seen that the fabricated sensor based on PDDA-AuPtAg/RGO displayed noticeably improved properties, including a wider linear range and a lower detection limit for H_2_O_2_. These improvements could be attributed to the excellent synergetic effects of the trimetallic alloy and the larger working surface provided by the PDDA-capped RGO nanosheets.

#### 3.4.3. Interference Immunity, Repeatability, and Stability

**Figure 5:**
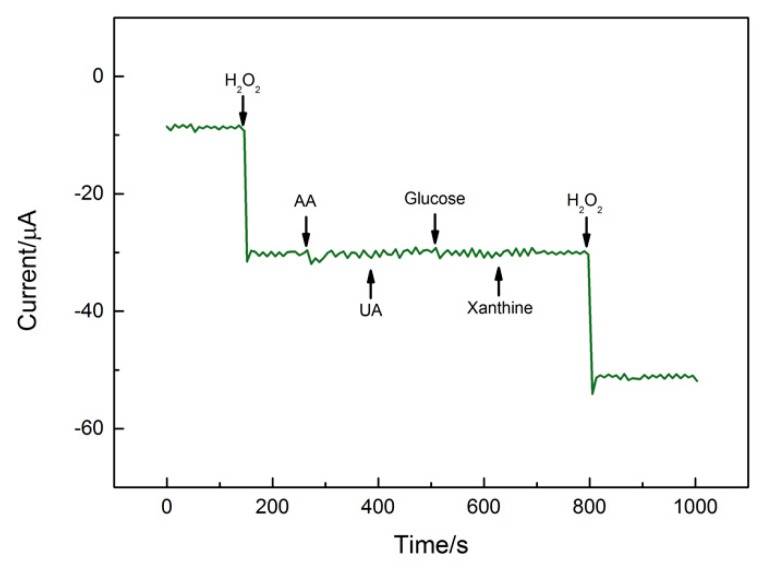
Amperometric i-t response of PDDA-AuPtAg/RGO/GCE upon the successive addition of H_2_O_2_ (0.1 mM), ascorbic acid (AA; 1 mM), glucose (1 mM), uric acid (UA; 1 mM), xanthine (1 mM), and H_2_O_2_ (0.1 mM) to 0.1 M PBS (pH 7.0) at an applied potential of 0.13 V with stirring.

Specificity, reproductivity, and long-term stability are important indicators when evaluating the accuracy of the designed electrochemical sensors. An anti-interference study was performed with typical probes, including ascorbic acid, glucose, uric acid, and xanthine. As shown in Figure 5, variations compared with the current changes caused by H_2_O_2_ caused by the interference were almost non-existent (less than 2%), indicating that the constructed sensor had admirable selectivity and specificity.

To investigate the stability, the fabricated sensor was tested periodically. After storage at 4 °C for one month, 92% of the initial current response of the as-prepared electrodes remained, illustrating acceptable long-term stability. Five electrodes were utilized under optimal conditions for exploring the reproducibility. As shown in Figure S4, favorable reproducibility can be observed with a relative standard deviation (RSD) of 2.45%.

### 3.5. In Situ Monitoring of H_2_O_2_ Released From Living Cells

**Figure 6:**
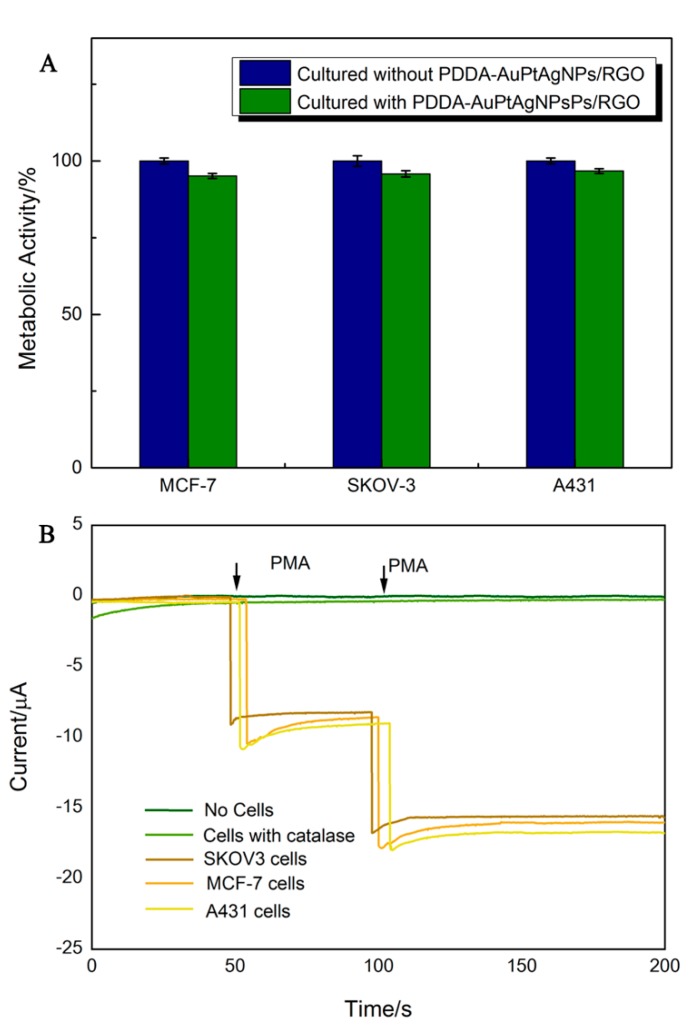
(**A**) Effect of PDDA-AuPtAg/RGO on the metabolic activity of SKOV3, MCF-7, and A431 cells. (**B**) Amperometric response of PDDA-AuPtAg/RGO/GCE upon the addition of 10 μL phorbol-12-myristate-13-acetate (PMA) (1 μg/mL) in the presence of cells with or without 300 U/mL catalases at the applied potential of 0.13 V.

The excellent performance of the PDDA-AuPtAg/RGO sensor provided the opportunity to explore its application in monitoring H_2_O_2_ in living cancer cells. It is well proven that H_2_O_2_ is a stable ROS, which is of great importance in various biological processes, and many disorders like cancer result from the unbalanced metabolism of ROS [47]. The dynamic process of releasing H_2_O_2_ upon oxidative stress was monitored with the as-prepared PDDA-AuPtAg/RGO/GCE after adding phorbol myristate acetate (PMA) to SKOV3, MCF-7, and A431 cell lines. PMA is a well-known stimulus for triggering H_2_O_2_ production to mimic oxidative metabolism in vivo. After stimulation of PMA, respiratory burst will occur and H_2_O_2_ will be the end product released from cells through fusion pores [48,49]. Determining the cytotoxicity of the obtained materials PDDA-AuPtAg/RGO was completed by MTT assays before the electrochemical measurements. As shown in Figure 6A, MCF-7, SKOV3, and A431 cells maintained 95.1%, 95.8%, and 96.7% viability, respectively, after 12 h incubation with PDDA-AuPtAg/RGO, suggesting the favorable biocompatibility of the biosensor.

Figure 6B exhibits amperometric response curves for PDDA-AuPtAg/RGO/GCE in the presence of SKOV3, MCF-7, and A431 cells triggered by the addition of PMA (10 μL, 1 μg mL^−1^), after which the currents changed significantly, indicating H_2_O_2_ released from these cancerous cell lines was reduced on the PDDA-AuPtAg/RGO modified electrode in real-time. PBS buffer without cells and cells with catalase were used as the control. There was no current response in the absence of cells and the current remained almost constant in the presence of 300 U/ml catalase (an H_2_O_2_ scavenger) in the PBS buffer. These results reveal that the current responses can undoubtedly be ascribed to the reduction of H_2_O_2_ released from the cancerous cells upon oxidative stress [50]. After the stimulation by 10 μL PMA, the current change in the SKOV3 cells was around 8.27 μA, and according to the calibration curve in Figure 4B, the amount of H_2_O_2_ was calculated as ~1.98 μM. Similarly, the current changes for the MCF-7 and A431 cells were 8.36 and 8.4 μA, respectively, which correspond to ~2.21 and ~2.34 μM H_2_O_2_ released from the cells. To confirm the accuracy, an Amplex Red Hydrogen Peroxide Assay kit (Molecular Probes) was used according to the manufacturer’s instructions (Table S2), which revealed a relative deviation between the two methods of less than 2.5%. These results suggest the excellent practicality of the proposed biosensor and its huge potential for the investigation of the mechanisms in ROS-enhanced disorders.

## 4. Conclusions

An ultrasensitive electrochemical sensor based on PDDA-capped AuPtAg/RGO was designed for the dynamic monitoring of intracellular H_2_O_2_. The synergetic effects of the trimetallic alloy ensured exceptional electrochemical catalysis toward H_2_O_2_ and definitely accelerated electron transfer, while the PDDA–RGO nanosheets employed as a supporting framework effectively enlarged the active surface area. The proposed sensor demonstrated admirable performance toward H_2_O_2_ detection in the concentration range from 0.05 μM to 5.5 mM with a low detection limit of 1.2 nM (signal-to-noise ratio of 3). In addition, the developed sensor was capable of real-time H_2_O_2_ measurements in three cancerous cell lines, thereby indicating its huge potential for the in-depth study of the role of ROS in tumor formation and the screening of cancer at an early stage.

## Supplementary Materials

The following are available online at https://www.mdpi.com/1424-8220/20/1/71/s1, Figure S1. EDX spectrum of PDDA-AuPtAg/RGO; Figure S2. Effects of concentration of PDDA-AuPtAg/RGO nanocomposite (0.3, 0.5, 0.8, 1.0, 1.5 mg/mL) and the deposition volume of PDDA-AuPtAg/RGO nanocomposite (3, 4, 5, 6, 7 μL) in 0.1 mM H_2_O_2_ and 0.1 M PBS (pH 7.0); Figure S3. The effect of pH on the reduction peak current of 5 mM H_2_O_2_ in a 0.1 M PBS at a scan rate of 50 mV/s; Figure S4. Column graph of CV signals of 0.1 mM H_2_O_2_ in 0.1 M PBS (pH 7.0) at five different electrodes prepared under the same conditions; Table S1 comparison of the reported sensors for H_2_O_2_ determination; Table S2. Comparison on detection of H_2_O_2_ released from living cells (1 × 106) by different methods (n = 3).
