# Supplementary material for: A Non-Enzymatic Sensor Based on Trimetallic Nanoalloy with Poly (Diallyldimethylammonium Chloride)-Capped Reduced Graphene Oxide for Dynamic Monitoring Hydrogen Peroxide Production by Cancerous Cells
Source: Sensors (Basel). 2019 Dec 21;20(1):71. doi: 10.3390/s20010071 (PMC6982804; doi:10.3390/s20010071)
Supplement: Supplementary file 1 [file sensors-20-00071-s001.pdf]

Supporting Information

A non-enzymatic sensor based on trimetallic nanoalloy with poly (diallyldimethylammonium chloride)-capped reduced graphene oxide for dynamic monitoring hydrogen peroxide production by cancerous cells

Jun Jiao, Meixin Pan, Xinran Liu, Binshuai Li, Jian Liu, Qiang Chen *

The Key Laboratory of Bioactive Materials Ministry of Education, College of Life Science, Nankai University, Weijin Road No. 94, Tianjin 300071, PR China

---

*Corresponding author:

E-mail address: qiangchen@nankai.edu.cn (Q. Chen).

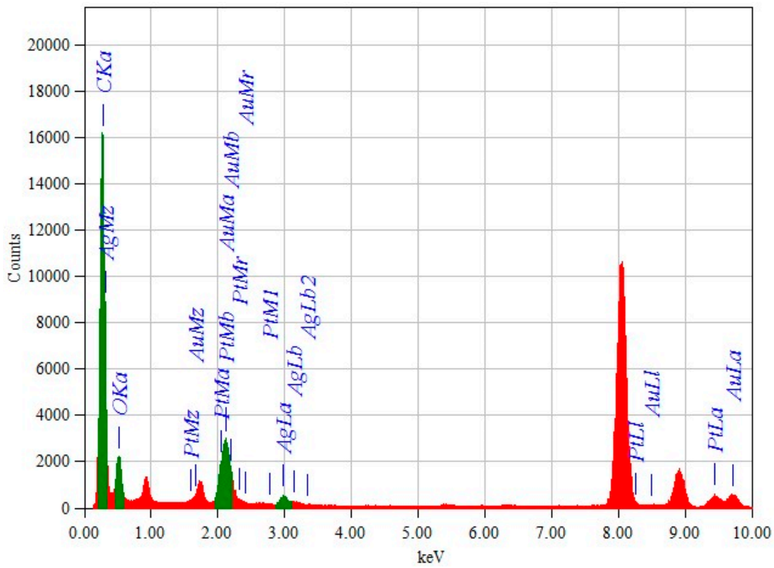

Figure S1. EDX spectrum of PDDA-AuPtAg/RGO

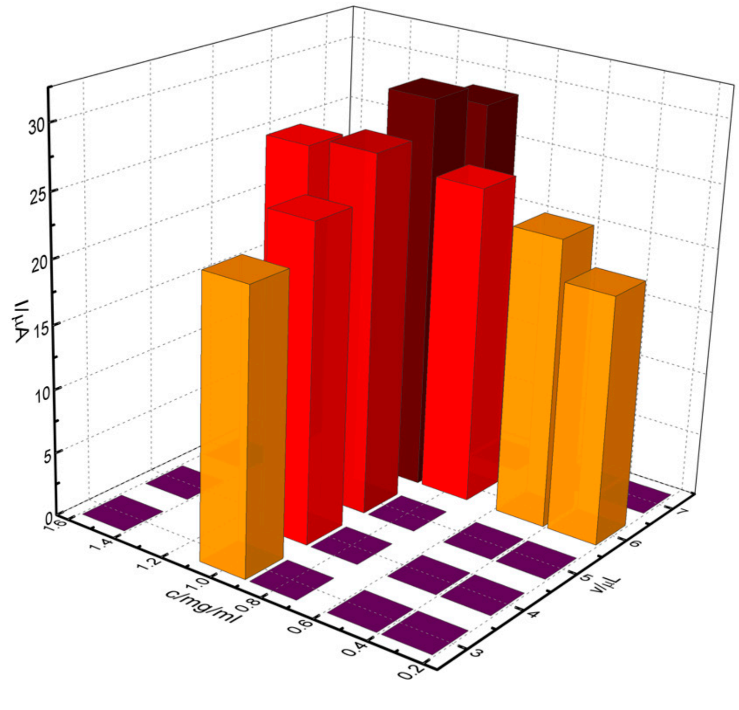

Figure S2. Effects of concentration of PDDA-AuPtAg/RGO nanocomposite (0.3, 0.5, 0.8, 1.0, 1.5mg/mL) and the deposition volume of PDDA-AuPtAg/RGO nanocomposite (3, 4, 5, 6, 7μL) in 0.1mM H_2O_2 and 0.1M PBS (pH 7.0).

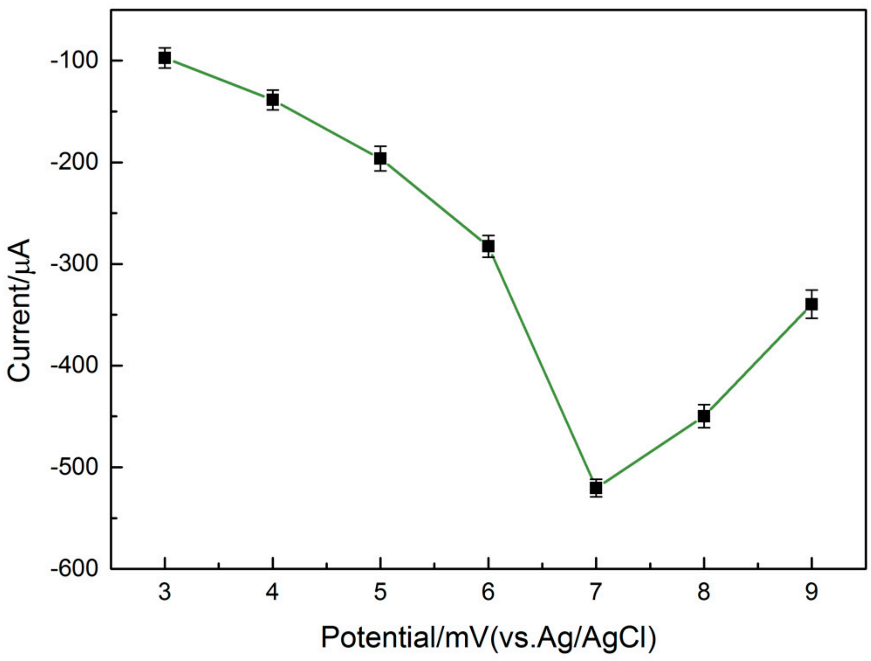

Figure S3. The effect of pH on the reduction peak current of 5 mM H_2O_2 in a 0.1 M PBS at a scan rate of 50 mV/s.

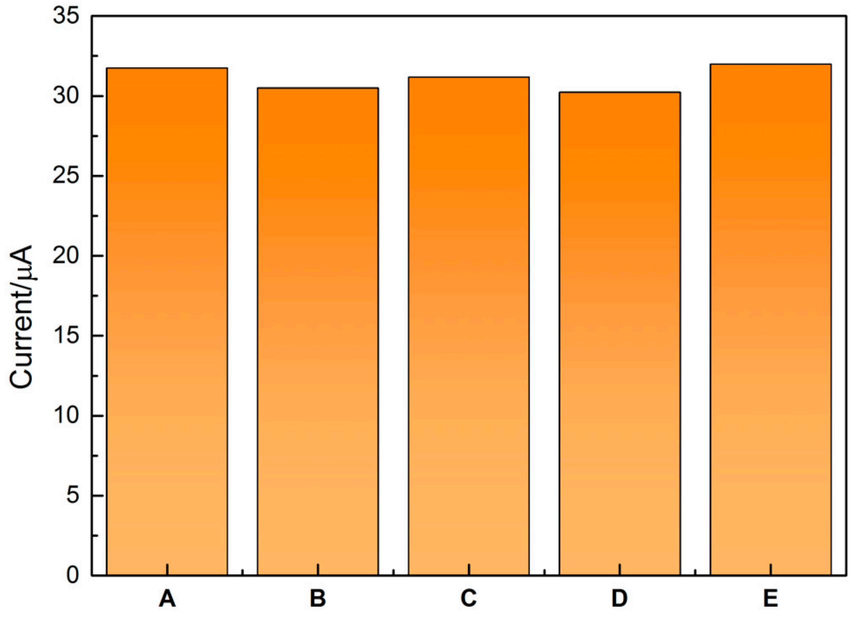

Figure S4. Column graph of CV signals of 0.1mM H_2O_2 in 0.1M PBS (pH 7.0) at five different electrodes prepared under the same conditions.

Table S1 Comparison of the reported sensors for H_2O_2 determination.

Materials	Detection range(μM)	LOD(μM)	Ref
Au/Carbon fiber	0.1–4300	0.05	[1]
CμF/Ag NPs-Naf	0.1–4300	0.05	[2]
Porous PtPd nanorods	0.02–50000	0.009	[3]
Hem@Au/rGO/CS	0.05-1000	0.0093	[4]
PtRu/3D Graphene	0.005-20	0.04	[5]
HRP-MIL-100(Cr)-B	0.5-3000	0.1	[6]
PDDA-AuPtAg/RGO	0.05-5.5	0.0012	This work

Table S2 Comparison on detection of H_2O_2 released from living cells (1×10^6) by different methods. (n=3).

Sample	Proposed sensor (μM)	Amplex Red Hydrogen Peroxide Assay kit (μM)	Relative deviation (%)
SKOV3	1.98	2.02	1.86
MCF-7	2.21	2.19	2.04
A431	2.34	2.31	1.36

[1] Y. Zhang, J. Xiao, Y. Sun, L. Wang, X. Dong, J. Ren, W. He, F. Xiao, Flexible nanohybrid microelectrode based on carbon fiber wrapped by gold nanoparticles decorated nitrogen doped carbon nanotube arrays: In situ electrochemical detection in live cancer cells, Biosensors and Bioelectronics, 100 (2018) 453-461.

[2] M. Gholami, B. Koivisto, A flexible and highly selective non-enzymatic H2O2 sensor based on silver nanoparticles embedded into Nafion, Applied Surface Science, 467 (2019) 112-118.

[3] S. Ge, W. Liu, H. Liu, F. Liu, J. Yu, M. Yan, J. Huang, Colorimetric detection of the flux of hydrogen peroxide released from living cells based on the high peroxidase-like catalytic performance of porous PtPd nanorods, Biosensors and Bioelectronics, 71 (2015) 456-462.

[4] W. Wang, H. Tang, Y. Wu, Y. Zhang, Z. Li, Highly electrocatalytic biosensor based on Hemin@ AuNPs/reduced graphene oxide/chitosan nanohybrids for non-enzymatic ultrasensitive detection of hydrogen peroxide in living cells, Biosensors and Bioelectronics, 132 (2019) 217-223.

[5] B. Bo, T. Zhang, Y. Jiang, H. Cui, P. Miao, Triple Signal Amplification Strategy for Ultrasensitive Determination of miRNA Based on Duplex Specific Nuclease and Bridge DNA-Gold Nanoparticles, Anal Chem, 90 (2018) 2395-2400.

[6] H. Dai, W. Lü, X. Zuo, Q. Zhu, C. Pan, X. Niu, J. Liu, H. Chen, X. Chen, A novel biosensor based on boronic acid functionalized metal-organic frameworks for the determination of hydrogen peroxide released from living cells, Biosensors and Bioelectronics, 95 (2017) 131-137.
